# Duration and dosing of Proton Pump Inhibitors associated with high incidence of chronic kidney disease in population-based cohort

**DOI:** 10.1371/journal.pone.0204231

**Published:** 2018-10-17

**Authors:** Antonio Rodríguez-Poncelas, Maria A. Barceló, Marc Saez, Gabriel Coll-de-Tuero

**Affiliations:** 1 Research Support Unit, University Institute of Research in Primary Care JordiGol (IdIAPGol), Girona, Spain; 2 Research Group on Statistics, Econometrics and Health (GRECS), University of Girona, Girona, Spain; 3 CIBER of Epidemiology and Public Health (CIBERESP), Madrid, Spain; 4 Department of Medical Sciences, University of Girona, Girona, Spain; Hospital Universitario de la Princesa, SPAIN

## Abstract

**Background:**

Proton Pump Inhibitors (PPIs) have been associated with chronic kidney disease (CKD). Our objective was to quantify the association between PPI use and incident CKD in a population-based cohort.

**Methods and findings:**

We used a population-based retrospective cohort, including people aged 15 years or over, between January 1, 2005 and December 31, 2012. PPI use was measured in a follow-up session by recording prescriptions. Incident CKD was defined as an estimated glomerular filtration rate < 60 ml/ min/1.73 m^2^ and/or urinary albumin level to creatinine level ≥ 30 mg/g, in two or more determinations over a period of at least 3 months of the follow-up. Proton Pump Inhibitor use was associated with incident CKD in analysis adjusted for different clinical variables (Hazard Ratio (HR) 1.18; 95% CI 1.04–1.51) in individuals who used PPI in the basal visit (HR 1.37; 95% CI 1.25–1.50) and in those who started to use PPI during the follow-up. High doses of PPI increased the risk of incident CKD (HR 1.92; 95%CI 1.00–6.19) for any type of exposure to PPIs (HR 2.40; 95%CI 1.65–3.46) and for individuals who used high doses throughout the follow-up. This risk of incident CKD increased after three months’ exposure to PPIs, (HR1.78; 95% CI 1.39–2.25) between the third and sixth months and (HR 1.30; 95%CI 1.07–1.72) after the sixth month.

**Conclusions:**

PPI use is associated with a higher risk of incident CKD. This association is greater for high doses and becomes apparent after three months’ exposure.

## Introduction

Chronic kidney disease (CKD) affects approximately 9.2% of adults in Spain[1]. While some risk factors are known—diabetes, hypertension, age, a family history of CKD[2,3]—the excessive prescription of Proton Pump Inhibitors (PPIs) and polypharmacy in the elderly could have contributed to the increase in CKD in the population[4,5].

PPIs are the most commonly used drugs to medically treat gastrointestinal conditions related to acidity, such as gastroesophageal reflux disease and the prevention and cure of gastro duodenal ulcers[6,7], and they are one of the most used drugs in the world[4]. PPI use has increased considerably in Spain in recent years [8,9]. They are commonly prescribed for conditions for which their use brings little benefit[6] and the inadequate prescription of PPIs in hospital discharges (for instance, itis commonly prescribed for stress ulcer prophylaxis for hospitalized noncritical ill patients without an appropriate indication) is relatively frequent[10,11], especially for elderly patients[12]. The general use of PPIs as a treatment in clinical practice means that although the health risks associated with their use is low, this could have serious consequences due to the high number of patients using these drugs[13].

Causes of acute interstitial nephritis (AIN) include drugs, autoimmune diseases, and infections. The most common aetiology of AIN are drug-induced diseases. Several studies have identified an association between PPI use and acute kidney injury (AKI),and most AKI events were identified specifically in the form of acute interstitial nephritis (AIN)[14–17]. Although they are well tolerated, several studies have linked the use of PPIs to AIN[14,15] and which can potentially lead to chronic kidney disease (CKD)[16,17]. Recently, Lazarus et al.,[18], Xie et al.,[19] and Arora et al.,[20], showed that PPIs use is associated with a higher risk of chronic kidney disease (CKD).

In this article, we intend to provide evidence on the association between PPI use and the incidence of chronic kidney disease and to find out if there is a relationship between dose and exposure time. These two areas are the main methodological contributions of our work. First, we used a retrospective cohort from the general adult population (≥ 15 years old), in which we did not exclude participants because of age or any other reasons. Second, we use statistical methods that allow us to minimize those methodological problems not contemplated in other studies, such as the existence of non-proportional risks, individual heterogeneity, both constant and time-varying, and the presence of unobservable confounders.

## Methods

### Ethical considerations of the study

The data for this study came from an anonymised clinical administrative database and only the lead researcher, where necessary, had access to the identity of each individual. This study has also been revised and approved by the Ethics and Clinical Research Committee of the Institute of Health Care (IAS).

### Study design and setting of the IAS study

The Catalan public healthcare system guarantees universal and free healthcare to all its citizens. This system is characterized by a division between healthcare funding (from the Catalan public budget) and the provision and management of the healthcare services. Catalonia is divided into seven health regions of which a Basic Area of Health (ABS, *‘Àreas Bàsiques de Salut’*, acronym in Catalan) is a territorial division. All residents in an area covered by an ABS are ‘assigned’ to the provider responsible for that particular ABS. The Institute of Health Care (IAS, *‘Institut d’Assistència Sanitària’* in Catalan), a primary healthcare service provider manages all the ABSs that provide healthcare to the region of ‘*La Selva Interior’*, Girona, Spain (ABS Anglès, ABS Breda-Hostalric and ABS Cassà de la Selva). *La Selva Interior* and *La Selva Marítima* form the *La Selva* ‘comarca’ (equivalent to a county) (further details can be found elsewhere[21]).

We used a population-based retrospective cohort composed of individuals who had made use of the primary healthcare services offered by any one of the three ABSs managed by the IAS. The study population included people aged 15 years or over who, between January 1, 2005 and December 31, 2012, made use of the public primary healthcare services offered by the primary healthcare centres which are managed by the Institute of Health Care (IAS, ‘Institutd’AssistènciaSanitària’ in Catalan, Girona, Spain).

All the data were obtained from clinical records and stored following a standardized protocol in the (centralized) IAS information system. The data for this study were drawn from that information system conforming to a clinical-administrative database, which contains anonymized information about patients that encompasses medical diagnoses, prescriptions, investigations, and referrals to secondary care and hospital discharge reports.

### Participants in the IAS study

**Fig 1:**
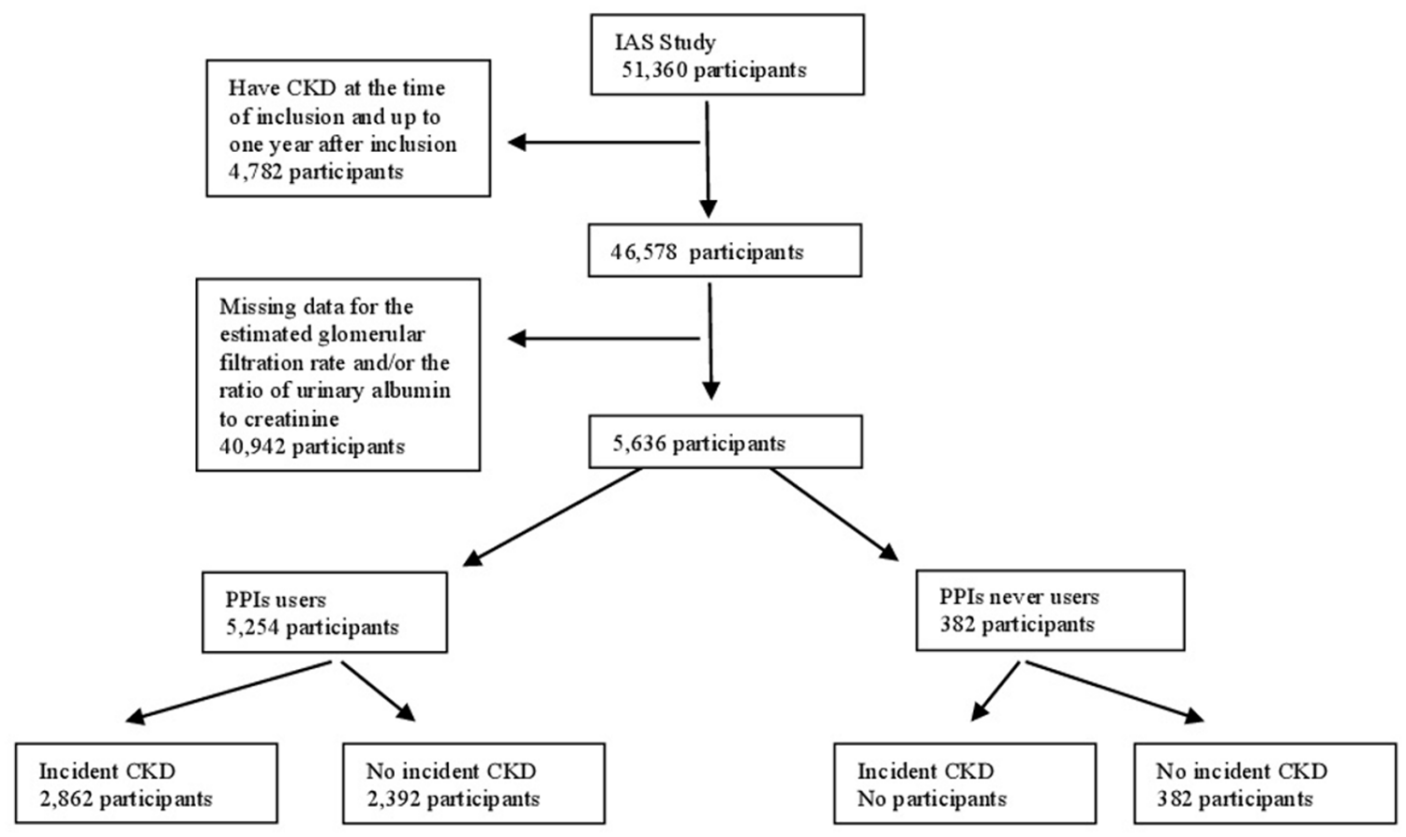
Flow diagram.

We included 51,360 participants aged 15 years or older (Fig 1). For each participant we obtained, in the first year of the follow-up (i.e. 2005), all the available measures of the estimated glomerular filtration rate (eGFR) and of the ratio of urinary albumin level to creatinine level (UACR). We estimate glomerular filtration rate (eGFR) using the CKD-EPI (Chronic Kidney Disease Epidemiology Collaboration)[22].

First, we excluded pre-existing CKD patients during the first year of the follow-up (n = 4,782 participants).

Our final sample was composed of participants who had, during the follow-up period (i.e. 2005–2012), some measure of eGFR and/or of UACR (n = 5,636 participants).

### Measurement of incident CKD

We defined incident CKD with two separate outcomes: a) an eGFR< 60 ml/ min/1.73 m^2^ and/or b) UACR ≥ 30 mg/g, in two or more determinations in a period of a minimum of 3 months. In fact, we were interested not only in the occurrence of CKD, but, above all, in the time elapsed from inclusion in the cohort until the onset of CKD.

Participants who died before developing CKD, could not be followed during the entire follow-up period, or did not have CKD before December 31, 2012, were considered (right) as censored.

### Measurement of PPI and other covariates

The use of PPIs was measured during the follow-up by recording the prescription in the medical records. PPIs available were: omeprazole, esomeprazole, lansoprazole, pantoprazole and rabeprazole. We considered exposure to PPIs as beginning on the date of the PPI prescription and ending after its calculated duration, including any consecutive prescriptions. We calculated the duration of each PPI prescription by using the number of treatment days recorded by the general practitioner or by dividing the total prescription quantity by the numeric daily dose prescribed on each prescription. Therefore, the exposure time to PPI was the total during the follow-up period, not necessarily continued over time. The duration of the PPI prescription was categorized as indicated by the Spanish Agency of Drugs and Sanitary Products[23], as less than a month, 1–3 months, 3–6 months, 6–12 months, 12–24 months, and more than 24 months. In addition, we categorized the PPI dose as either standard (20 mg for omeprazole, esomeprazole and pantoprazole, 15 mg for lansoprazole, and 10 mg for rabeprazole) or high (21–40 mg for omeprazole, esomeprazole and pantoprazole, 16–30 mg for lansoprazole, and 11–20 mg for rabeprazole).

We adjusted for the following covariates: i) sex (0 male—reference category -, 1 female), ii) country of birth (1 Spain, 0 other—reference category) and iii) age. We categorized the age variable (0 being under 70 years—reference category -, 1 being 70 years or older), since the relationship between our response variable and age was clearly non-linear, with the cutoff point at 70 years.

iv) Medical conditions: diagnosed hypertension. Subjects with at least two blood pressure readings of ≥ 140 and/or 90 mmHg, a previous diagnosis of hypertension or those receiving treatment with anti-hypertensive medication were considered to have hypertension. High blood pressure was defined as having had at least two blood pressure readings taken in the doctor’s surgery of between 130–139 and/or 85–89 mmHg and/or being diagnosed hypertension. The criteria of the American Diabetes Association (ADA) was used for the diagnosis of type II diabetes (DM2)[24]: clinical symptoms plus a random glucose ≥200 mg/dl, two fasting plasma glucose ≥ 126 mg/dl, two determinations of HbA1c ≥ 6,5% or two 2-hour plasma glucose levels ≥ 200 mg/dl. Patients with a previous diagnosis or those being treated with antihyperglycemics were also considered to have DM2. Impaired glucose tolerance was defined as basal glucose levels ≥110 mg/dL. Obesity was defined as a body mass index > 30 kg/m^2^, low high density lipoproteins (HDL) (in men<40mg/dL, in women<50mg/dL), and hypertriglyceridemia (≥150 mg/dL). Metabolic syndrome (MS) was also considered. A subject from the cohort was considered to have MS if they had three or more of the five possible conditions concurrently[25,26], i.e. a diagnosis of diabetes or intolerance to glucose, a diagnosis of hypertension or high blood pressure, and/or dyslipidaemia i.e. low high density lipoprotein levels(HDL), hypertriglyceridemia, and/or obesity i.e. BMI> 30 kg/m^2^.

v) Number of chronic diseases (other than renal diseases and metabolic disorders), included ischemic heart disease, heart failure, atrial fibrillation, peripheral arteriopathy, left ventricular hypertrophy, cerebral vascular accident, dementia, retinopathy, macular edema and cancer.

vi) Smoking status (0 non-smoker—reference category, 1 smoker, 2 former smoker). Alcohol consumption (0 Non-drinker—reference category, 1 alcoholic, 2 ex-alcoholic).

vii) Treatments: antihypertensive treatment, antidiabetic treatment, anti-hypolemics and NSAIDs.

### Statistical analysis

The models were estimated using only those participants with measures of eGFR and/or UACR available during the follow-up.

Baseline characteristics of all patients (i.e. PPI never users and PPI users, stratified in PPI users at baseline and PPI users only in the follow-up) were summarized by means and standards deviations (quantitative variables) and by proportions (qualitative variables). The bivariate associations between PPI use (stratified as above) and CKD were assessed with chi-squared tests.

**Fig 2:**
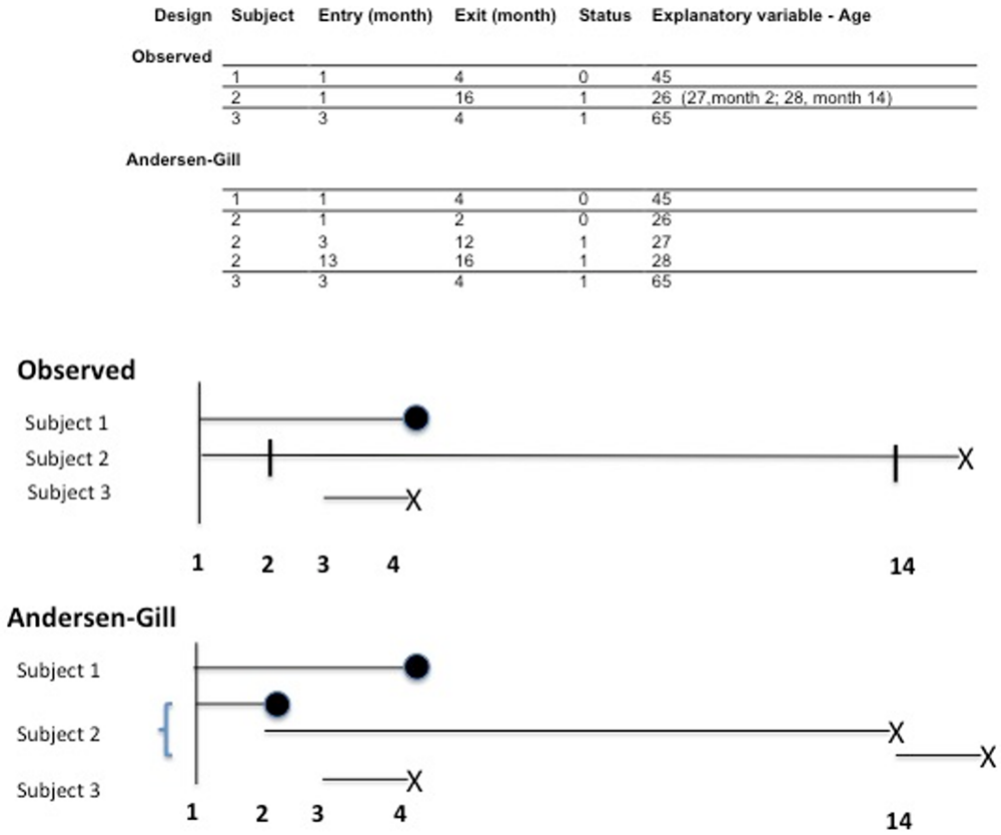
Example of the Andersen-Gill design.

In the multivariate analysis we performed survival analysis. We were interested not only in the risk of occurrence of CKD as a consequence of the use of PPI, but also over time, i.e. from inclusion in the cohort, which takes place from when PPI use began and the occurrence of CKD. There was a problem with both the exposure variables, i.e. the use of PPI, and most of the covariates being time dependent. Under these conditions, the risks (of occurrence of CKD) were not proportional. Another important problem is that of delayed entry. The subjects in our study did not enter the study at the beginning of follow-up, but rather throughout the follow-up. As a consequence, the times-to-event were not a random sample from the population. These problems prevented us from using the Cox model. First, because the main assumption of the Cox model, also known as proportional risks, is not met. Second, because another important assumption the Cox model makes is that subjects are comparable, a fact that could not be met if the times-to-event sample was not random. In fact, failure to adjust for delayed entry can lead to biased estimates[27]. For all these reasons, we chose to use the Andersen-Gill (AG) model of multivariate survival analysis[28–30] instead. The idea, in addition to allowing delayed entry, is to divide each participant’s exposure time (up to the occurrence of CKD or censoring) into intervals (not necessarily equal) in which the risk would not change (and therefore would be proportional). To clarify the design implied by the AG model, we show a small example in Fig 2[30]. Consider three individuals. Two of them enter the study at time 1 and the third enters at time 3. All three, however, leave the study at different times, some with the event of interest (status equal to 1) and others without it having happened (status equal to 0, right censoring). The explanatory variable is time-dependent. However, it only changes its value in subject 2 (changes value twice). In the design considered by the AG model, individuals 1 and 3 have an interval, but individual 2 has 3 intervals. Note, also, that in subject 2 the event occurs before the end of its follow-up, a fact that could not be collected in a standard design. Now, in each of the intervals, the standard survival analysis could be used. Of course, in this case it should be borne in mind that observations (corresponding to the same participant) are not independent and appropriate estimation methods must be used. In addition, the AG model adjusts appropriately for the delayed entry.

In the model, in addition to the exposure variables (of PPI use), we adjusted for all the covariates indicated above. We also controlled for the presence of unobserved confounders by introducing into the regression two random effects. The first one captured the individual heterogeneity (also called frailty in survival analysis). That is to say, unobservable factors, specific to each participant, explanatory of the risk for which no information is available and which are constant in the follow-up period. In this case, we used a vector of Gaussian, zero-mean, unstructured, identically and independently distributed random variables. The second one captured contextual unobserved confounders Our hypothesis is that there could be contextual variables, for example socioeconomic variables (i.e. material deprivation), which, if not observed, could not be included in the model, thus producing spatial dependence which should be controlled. Therefore, after georeferencing the address of each participant, we included a vector of structured random variables (with a spatial Matérn structure) on an irregular mesh[31].

Now, we should consider that there could be unobserved explanatory factors of risk (called hazard in survival analysis), also specific to each participant, but that, unlike frailty (i.e. individual heterogeneity), varied over time. These factors, included in the so-called baseline hazard, are considered a nuisance in the standard Cox model. Here, however, we modelled the log baseline hazard as a piecewise constant function on small time intervals, and impose smoothness to penalise deviations from a constant[32].

Given the complexity of our model, we prefer to perform inferences using a Bayesian framework. This approach is considered the most suitable to account for model uncertainty, both in the parameters and in the specification of the models. Moreover, only under the Bayesian approach is it possible to model extra variability with relatively sparse data in some cases. Finally, within the Bayesian approach, specifying a hierarchical structure on the (observable) data and (unobservable) parameters, which are all considered as random quantities, is straightforward. We used penalising complexity (PC) priors[33]. These priors are invariant to reparameterisations and have robustness properties. In particular, we follow the Integrated Nested Laplace Approximation (INLA) approach[34], within a (pure) Bayesian framework.

Once the models were estimated, survival curves of the baseline hazard, stratified by use of PPI, were estimated using the Kalbfleisch-Prentice method[35], which is equivalent to the Kaplan Meier estimates when the weights are unity (as in our case). Survival curves were compared using the log rank test[36].

We controlled the presence of unobserved confounders by introducing two random effects into the regression (hence the term ‘mixed’ in the name of the regression), one controlling for individual heterogeneity and the other for contextual unobserved confounders.

All analyses have been done with the free software R (version 3.2.3)[37], available though the INLA library[38,34].

## Results

The final sample size was 46,541 individuals, 49.8% of whom were women. Of the total participants 31,246 did not use PPIs during the study period, 12,202 used PPIs in the basal visit and 3,093 began treatment with PPIs during the follow-up. Compared with nonusers, PPI users were older, a higher percentage were women and they exhibited greater obesity, hypertension, dyslipidaemia, type 2 diabetes, cardiovascular disease and took more antihypertensive medication, NSAIDs and statins (Table 1). Participants that used PPIs in the basal visit had lower eGFR values than individuals that began treatment with PPIs after the basal visit. The average age of the patients who developed CKD was 71.9 years (standard deviation 8.8).

**Table 1:** Baseline data of patients included in the cohort^[^^1^^]^.

Variables	n	All	n	PPI never users	n	PPI users at baseline	n	PPI users in follow-up (only)
**Age; Mean (SD)**	46,541	41.23 (20.375)	31,246	37.11 (20.22)	12,202	48.39 (18.11)	3,094	54.53 (16.51)
**Women; n (%)**	23,167	23,167 (49.8)	14,915	14,914 (47.8)	6,455	5,782 (47.4)	1,798	1,798 (58.1)
**Men; n (%)**	23,370	23,370 (50.2)	16,296	16,296 (52.2)	5,782	6,455 (52.7)	1,296	1,296 (41.9)
**BMI; Kg/m**^**2**^ **;mean (SD)**	9,815	26.33 (5.10)	9,815	26.33 (5.10)	0	0	0	0
**Tobacco [No smoker]**^**[**^^2^^**]**^**; n (%)**	46,578	46,578	31,247	31,247 (67.09)	12,237	12,237 (26.3)	3,094	3,094 (6.6)
**Tobacco. Smoker; n (%)**	4,287	4,287 (9.2)	2,545	2,545 (8.1)	1,430	1,430 (11.7)	312	312 (10.1)
**Tobacco. Past smoker; n (%)**	854	854 (1.8)	386	386 (1.2)	364	364 (3.0)	104	104 (3.4)
**Obesity; n (%)**	46,578	6,741 (14.5)	31,247	2,640 (8.4)	12,237	3,040 (24.8)	3,094	1,061 (34.3)
**Hypertension; n (%)**	46,578	6,897 (14.8)	3,1247	2,386 (7.6)	12,237	3,348 (27.4)	3,094	1,163 (37.6)
**Dyslipemia; n (%)**	20,137	7349 (36.5)	10,971	3,386 (30.9)	7,008	2,984 (42.6)	2,158	979 (45.4)
**Fasting glucose; mean (SD)**	20,435	92.75 (24.04)	11,197	90.08 (20.55)	7,063	95.91 (27.24)	2,175	96.27 (27.69)
**Type 2 DM; n (%)**	46,578	7,665 (16.5)	31,247	2,776 (8.9)	12,237	3,723 (30.4)	3,094	1,166 (37.7)
**UAER (mean (SD)**	2,155	16.32 (13.55)	668	17.06 (13.80)	1,022	16.49 (13.54)	465	14.89 (14.90)
**GFR (CKD-EPI); mean (SD)**	3,703	86.60 (119.38)	1,562	87.86 (103.32)	1,526	76.33 (65.77)	615	108.89 (187.46)
**Cardiovascular disease, n (%)**	5,636	1,244 (22.1)	1562	116 (7.4)	1243	402(32.3)	2831	726 (25.6)
**Microalbuminuria; n (%)**	2,155	730 (33.9)	668	246 (36.8)	1,022	349 (34.1)	465	135 (29.0)
**Antihypertensive drug; n (%)**	46,578	2,920 (6.3)	31,247	777 (2.5)	12,237	1,483 (12.1)	3,094	660 (21.3)
**Diuretic; n (%)**	46,578	1,898 (4.1)	31,247	468 (1.5)	12,237	960 (7.8)	3,094	470 (15.2)
**ACEI/ARB; n (%)**	46,578	1,152 (2.5)	31,247	282 (0.9)	12,237	617 (5.0)	3,094	253 (8.2)
**NSAID; n (%)**	46,578	9,997 (21.5)	31,247	4,573 (14.6)	12,237	3,865 (31.6)	3,094	1,559 (50.4)
**Statins; n (%)**	46,578	265 (0.6)	31,247	70 (0.2)	12,237	141 (1.2)	3,094	54 (1.7)
**PPI_baseline [No PPI 2005–2012]**^**[**^^2^^**]**^**; n (%)**	46,578	46,578	31,247	31,247 (67.09)	12,237	12,237 (26.3)	3,094	3,094 (6.6)
**No baseline PPI; n (%)**	12,237	12,237 (26.3)	0	0 (0)	0	0 (0)	3,094	3,094 (100)
**Baseline PPI; n (%)**	3,094	3,094 (6,6)	0	0 (0)	12,237	12,27b (100)	0	0 (0)

^1^ General adult population who did not have MRC at the time of inclusion and up to one year of inclusion

^2^ Reference category in square brackets

BMI: Body mass index; DM: Diabetes mellitus; UAER: Urinay albumin excretion rate; GFR: glomerular filtration rate; ACEI: angiotensin-converting- enzyme inhibitor; ARB: angiotensin receptor blocker; NSAID: Non-steroidal anti-inflammatory drug; PPI; Proton inhibition pump; cardiovascular disease: coronary heart disease, stroke, peripheral arterial disease, chronic heart failure.

Comparing the incidence of CKD in PPI users and nonusers (Table 2), individuals that developed CKD were shown to be older, with a higher incidence among women than men, and a greater prevalence of hypertension, diabetes and cardiovascular disease. They also took more antihypertensive medication, NSAIDs and statins. The comparison of individuals that developed CKD and those that did not among participants that used PPIs in either the basal visit or during the follow-up showed significant differences in all the variables studied. Significant differences were also observed among participants that developed CKD when comparing participants who were already taking PPIs in the basal visit and those that only took PPIs during the follow-up. No differences were observed in the participants that did not develop CKD during the follow-up between non-PPI users and those that already used PPIs in the basal visit, except for participants over 70 years old and their use of antihypertensive medication. Significant differences were observed, however, in patients that did not develop CKD during the follow-up when comparing individuals that had never used PPIs and those who had begun treatment with PPIs during the follow-up.

**Table 2:** Association between mode of proton inhibition pump use and incident chronic kidney disease.

Variables	Overall No CKD	Overall CKD	Overall p	PPI never user No CKD	PPI at baseline No CKD	PPI at baseline CKD	PPI at follow-up (only) No CKD	PPI at follow-up (only) CKD
**Age ≥70 years; n (%) n = 5,636**	991 (17.5)	1,299 (23.0)	<0.001	365 (6.5)	463 (8.2)*	818 (14.5)**	163 (2.9)§	481 (8.5)&/^
**Women n (%)**	580 (10.2)	1,626 (28.8)	<0.001	221(3.9)	247 (4.4)	1,057 (19.7)**	112 (2.0) §	569 (10.0)&/^
**Men n (%)**	2,194 (38.9)	1,236 (21.9)	<0.001	1,341 (23.7)	678 (12.0)	849 (15.06)**	175 (3.1) §	387 (6.9)&/^
**Hypertension; n (%), n = 4,567**	796 (17.4)	1,469 (32.0)	<0.001	336 (7.4)	348 (7.6)	976 (21.4)**	230 (5.0) §	493 (10.7)&/^
**Type 2 DM; n (%), n = 4,567**	871 (19.1)	1,596 (35.0)	<0.001	382 (8.4)	370 (8.1)	1,081 (23.6)**	119 (2.6) §	515 (11.2)&/^
**Cardiovascular disease, n (%), n = 5,636**	399 (7.1)	845 (15.0)	<0.001	116 (2.0)	93 (1.6)	309 (5.5)**	190 (3.4) §	536 (9.5)&/^
**Anti-hypertensive drugs, n (%), n = 5,636**	373 (6.6)	989 (17.5)	<0.001	116 (2.1)	197 (3.5)*	617 (10.9)**	60 (1.0) §	372 (6.6)&/^
**ACEI/ARB; n (%), n = 5,636**	160 (2.8)	428 (7.6)	<0.001	51 (0.9)	88 (1.5)*	275 (4.9)**	21 (0.4) §	153 (2.7)&/^
**Diuretic; n (%), n = 5,636**	226 (4.0)	708 (12.5)	<0.001	71 (1.3)	115 (2.0)*	431 (7.6)**	40 (0.7) §	277 (4.9)&/^
**NSAID; n (%), n = 5,636**	805 (14.3)	1,498 (26.5)	<0.001	355 (6.3)	315 (5.5)	893 (15.8)**	135 (2.4§¶	605 (10.7)&/^
**Statins; n (%), n = 5,636**	33 (0.5)	115 (2.0)	<0.001	7 (0.1)	21 (0.4)	77 (1.3)**	5 (0.1)	38 (0.6)&/^

CKD: chronic kidney disease; PPI: Proton Inbibition pump; DM: Diabetes mellitus; ACEI: Angiotensin-converting-enzyme inhibitor; ARB: Angiotensin receptor blocker; NSAID: Non-steroidal anti-inflammatory drug

n = 5,636 patients included with renal function data available

Significance level (Bonferroni correction): p < 0.01

* p<0.01 PPI never users (no CKD) vs PPI at baseline (no CKD)

** p<0.01 PPI at baseline (no CKD) vs PPI baseline (CKD)

^§^ p< 0.01 PPI never user (no CKD) vs PPI follow-up (no CKD)

^&^p< 0.01 PPI at baseline (CKD) vs PPI at follow-up (CKD)

^ p<0.01 PPI at follow-up (no CKD) vs PPI at follow—up (CKD)

After adjusting the results for different confounding factors, the risk of incident CKD during the follow-up in individuals who used PPI in the basal visit in relation to non-PPI users was 18% and 37% in participants that began taking PPIs during the follow-up. High doses of PPIs were shown to increase the risk of CKD by 92% for any type of exposure to PPIs and this risk increased evern more for the individuals who took high doses during the follow-up. The risk of incident CKD increased after three months’ exposure to PPIs: 78% between the third and sixth months and 30% after the sixth month (Table 3).

**Table 3:** Risk of incident chronic kidney disease with proton inhibition pumps use. Multivariate analysis in a cohort before correcting left censoring (only participants with available measurements of eGFR and/or UACR).

Strata	PPI users at baseline and/or follow-up (only)		PPI users at follow-up (only)	
---	Unadjusted HR (95%CrI)	Adjusted HR (95%CrI)	Unadjusted HR (95%CrI)	Adjusted HR (95%CrI)
PPI users vs never users	1.26 (1.02–1.64)	1.22 (1.08–1.57)	1.43 (1.01–1.73)	1.39 (1.15–1.61)
Dose. Standard	1.58 (1.06–5.41)	1.49 (1.04–5.11)	1.53 (0.93–5.71)	1.33 (0.90–3.31)
Dose. High	1.89 (1.06–5.97)	1.75 (1.05–5.56)	3.48 (1.23–5.26)	2.21 (1.15–3.76)
PPI exposure duration vs no exposure	---	---	---	---
< 1 month	1.30 (1.02–1.64)	1.30 (0.82–1.64)	1.17 (0.30–3.37)	1.20 (0.50–3.51)
1–3 months	1.17 (0.92–1.48)	1.16 (0.81–1.47)	1.16 (0.40–3.33)	1.22 (0.59–3.63)
3–6 months	1.45 (1.14–1.83)	1.42 (1.11–1.80)	1.51 (1.07–2.31)	1.42 (1.11–2.11)
> 6 months	1.19 (1.07–1.62)	1.16 (1.08–1.57)	1.73 (1.18–2.46)	1.61 (1.08–2.73)

Adjusted for: age, gender, impaired fasting glucose, type 2 diabetes, obesity, high-normal blood pressure, hypertension, low-HDL-cholesterol, hightriglycerides level, metabolic syndrome, chronic diseases, tobacco consumption, alcohol consumption, cardiovascular disease, antihypertensive treatment, hypoglucemiant treatment, hypolipemiant treatment, non-steroidal antiinflammatory drugs, other countries origin.

Table 4 shows the variables related to incident CKD in participants that used PPIs compared with nonusers. Age, impaired fasting glucose, type 2 diabetes, high-normal blood pressure, hypertension, triglycerides, cardiovascular disease and originating from other countries were associated with a greater prevalence of CKD in participants that used PPIs.

**Table 4:** Variables related to incident chronic renal disease in participants that used proton inhibition pump compared with nonusers. Multivariate analysis. Table 4 shows the variables related to incident CKD in participants that used PPIs compared with nonusers.

Variables	HR (95% CredibiIity Interval)
Gender (women)	1.04 (0.93–1.16)
Age ≥70 years	2.39 (2.14–2.67)
Impaired fasting glucose	1.19 (1.04–1.35)
Type 2 DM	1.43 (1.25–1.64)
High-normal BP	1.81 (1.62–2.03)
Hypertension	1.17 (1.04–1.32)
Cardiovascular disease	1.06 (1.00–1.13)
Triglycerides	1.35 (1.15–1.58)
Tobacco (yes)	1.11 (0.88–1.4)
Alcohol consumtion (yes)	0.67 (0.38–1.17)
Anti-hypertensive treatment	1.001 (1.000–1.002)
Hypoglucemiant treatment	1.001 (1.000–1.002)
Non-steroidal anti-inflammatory drugs	0.99 (0.99–1.00)
Other countries	**1.32 (1.14–1.51)**

Adjusted for: PPI use, dose of PPI, exposure duration and number of expositions at PPI.

HR: Hazard ratio; DM: Diabetes Mellitus; PPI pump.

**Fig 3:**
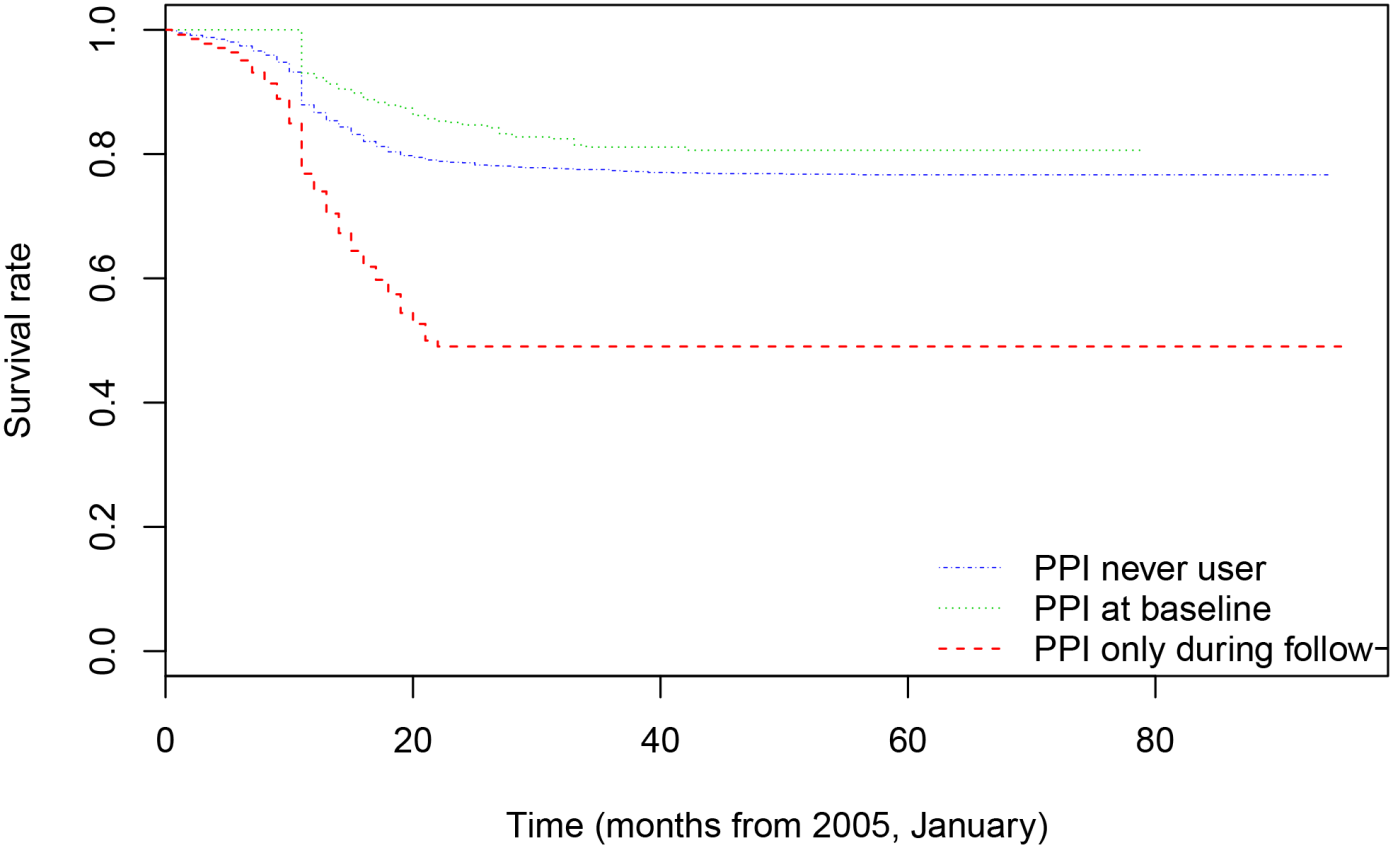
Chronic kidney disease survival rate in proton inhibition pump never exposed and exposed subjects (Andersen-Gill survival model). p-values of the log-rank tests: PPI never user vs. PPI at baseline p<0.001; PPI at baseline vs. PPI only during follow-up p<0.001; PPI never user vs. PPI at baseline and/or during follow-up p<0.001.

Fig 3 shows the survival curves of the baseline hazard (estimated from the survival models) stratified by the use of PPI. Although there were statistically significant differences between all curves, we found the greatest differences between those who did not use PPI during the follow-up (PPI never users) and those who did (PPI users at baseline, PPI users both, at baseline and during the follow-up). Note that the risk of developing CKD appears before the year of onset of exposure. However, CKD occurred much earlier in those participants who were exposed to PPIs at some time during follow-up and/or at baseline. Thus, at 10 months after exposure, while 7% of those who used PPI only during follow-up and also of those who used PPI at baseline developed CKD, 22.6% of the participants who used PPI at baseline and during the follow-up developed CKD. The risk of developing CKD stabilized approximately 22 months after exposure to PPI. At that time, 48.3% of PPI users at baseline and during follow-up, 21.4% of PPI users at baseline and 14.9% of PPI users only during follow-up, developed CKD.

## Discussion

In this retrospective community-based cohort of 46,541participants, and once we had adjusted for several potential confounding variables, including demographics, socioeconomic status, clinical measurements, prevalent comorbidities, and concomitant use of medications, we found that baseline, and follow-up use of PPIs was independently associated with a 18% to 37% higher risk of incident CKD, respectively. Furthermore, this risk increased considerably with the use of high doses of PPIs and with prolonged durations of exposure to them.

The association between PPI use and CKD was more pronounced in patients taking higher doses of PPI. Twice-daily PPI dosing was associated with a 15% higher risk than once-daily dosing[18]. The results show a graded association between duration of exposure and risk of renal outcomes. That said, the association seems to weaken in those exposed for more than 720 days, which is most likely a reflection of a survivorship bias, those remaining in the cohort are likely resistant to the effect of PPI on renal outcomes[18–20] The mechanisms of the associations between long duration or high dose PPI use causing more CKD are not clear. It is speculated that undiagnosed, unrecognized and partial recovery from PPI induced AIN could prime the kidney to develop subsequent AKI or CKD among PPI users or the existence of unrecognized AKI or chronic latent renal injury.

The risk of developing CKD appears before the year of onset of exposure to PPI and stabilized approximately 22 months after exposure. This risk is significant from the third month of exposure, be it isolated or accumulated. However, CKD occurred much earlier in those participants who were exposed to PPIs at some time during follow-up and/or at baseline, probably due to a selection bias, as participants that used PPIs in the basal visit could tolerate PPIs better and did not develop CKD.

PPIs are considered highly effective for the treatment of gastroesophageal reflux disease, acid-related disorders, as well as preventing and treating peptic ulcer disease and the effects of glucocorticoid or non-steroidal anti-inflammatory drugs. Although PPIs have been approved as safe by the Food and Drug Administrationonly for short-term treatment, they are frequently prescribed for even minor indigestion, leading to chronic use. In addition, PPIs have been prescribedunnecessarily as much as two-thirds of the time[39].

Since its introduction to the market, proton-pump inhibitor (PPI) utilization has increased rapidly and PPIs are among the most widely-used medication, in both prescription and over-the-counter sales.

The mechanisms of the associations between PPI use and acute kidney injury (AKI) could be through acute interstitial nephritis (AIN). Most AKI events were identified specifically in the form of AIN, which has been suggested by multiple studies as having an association with PPI exposure[14–17,40–42] and might be a cell-mediated idiosyncratic immune response[43],a class effect, as all PPIs could cause AIN. Several studies have attempted to estimate the incidence rate and relative hazards of the development of CKD, both in survivors of AKI and compared in populations without AKI. According to a meta-analysis[44], patients who survive AKI have a greater risk of CKD, ESRD, and other adverse outcomes compared with patients without AKI after adjustment for several important confounding variables. The hazard ratio for developing CKD following an episode of AKI was 8.8 (95% confidence interval: 3.1–25.5)[45]. Yang *et al*., recently assessed the risk of AKI in patients taking PPIs and observed a significant association between PPI use and a 1.61-fold increased risk of AKI[46].

The reasons why AKI would increase the risk of CKD, ESRD, and other adverse outcomes remain unknown. It is hypothesized that rarefaction of peritubular capillaries represents a critical event, following ischemic injury, that permanently alters renal function and predisposes patients to the development of chronic renal disease[43].

In patients who recovered renal function after AKI, observational studies have shown an association between AKI, including mild cases, and the subsequent development of CKD, an increased long-term risk of end-stage renal disease (ESRD), and excess mortality[45,47]. Even patients whose serum creatinine returns to baseline following an AKI episode have the possibility of progressing to CKD. Thus, despite the fact that AKI is typically reversible in nature, on the basis of serum creatinine concentrations there may be subclinical renal and extra-renal damage that persists and mediates these adverse outcomes. Sometimes recovery from acute ischemia-reperfusion injury is not complete, compromises sodium homeostasis, and predisposes hypertension and chronic renal disease[48].

AKI and CKD share common risk factors and disease modifiers. When AKI occurs without pre-existing kidney disease, CKD still may develop. Conversely, the presence of CKD is an important risk factor for the development of AKI. Either AKI or CKD is associated with an increased risk of death and may result in complications such as cardiovascular disease, progressive decreases in kidney function, diminished quality of life, and the development and progression of disability[49]. Elderly individuals with AKI, particularly those with previously diagnosed CKD, are at significantly increased risk for ESRD, suggesting that episodes of AKI may accelerate progression of CKD[50].

Similar results to ours were shown recently by Lazarus *et al*.[18], Xie *et al*.[19] and Arora *et al*.[20], although with considerable differences in the cohorts. We included participants aged 15 years or older in a cohort that was representative of the general population and we were interested not only in the occurrence of CKD, but also in the time that elapsed between inclusion in the cohort and the onset of CKD. Considering that PPI use and most of the covariates are time dependent and that the risks of CKD are not proportional, we used the Andersen-Gill (AG) model of multivariate survival analysis, dividing each participant’s exposure time (up to the occurrence of CKD or censoring) into intervals (not necessarily equal) in which the risk would not change (and therefore would be proportional). In this way, the standard survival analysis could be used in each of the intervals. In Arora *et al*., cohort PPI users had less cardiovascular comorbidity and it was found that younger individuals were more likely to develop CKD associated with PPI use[20]. It is possible that the prevalence of CKD among the elderly is high with or without PPI, but in the younger population the prevalence of CKD without PPI use is quite low, thus making the prevalence of CKD associated with PPI use more significant.

This study has several strengths and limitations that deserve some comment. In our analyses, we considered drug exposure as PPI prescription. However, since PPI is available over the counter in Spain, it is possible that some individuals in this cohort may have obtained and used PPI without prescription and subsequently this use would not have been recorded. That said, this is unlikely as in our health system it is usual to go through the primary healthcare centres for easy access to and funding of these drugs. Nevertheless, this risk must not be underestimated. Participants who are prescribed PPIs may be at higher risk of CKD for reasons unrelated to their PPI use. PPI-users in the IAS cohort were more likely to be obese, have a diagnosis of hypertension, dyslipidaemia, diabetes mellitus, cardiovascular disease, and took more concomitant medication. Furthermore, we also did analyses to resolve this potential bias, we performed an adjustment for multiple confounders, including BMI, hypertension, dyslipidaemia, diabetes mellitus, cardiovascular disease and concomitant medication use, comparing PPI users directly with nonusers. Each of these sensitivity analyses showed a consistent association between PPI use and a risk of CKD. We had no measure of eGFR or UACR for 88% of the participants and the majority of these were probably younger and had less risk of developing CKD. Determinations of eGFR and UACR in the young, healthy population are unusual in our context. These individuals with missing data were left censored. We did not know how many of these participants would develop CKD during the follow-up. The analysis of the sample before correcting left censoring and after, showed that the results were similar. We could not assess H2-receptor antagonists’ use as the active comparator because in this cohort few participants were taking H2-receptor antagonists.

Notable strengths of our study include a large, representative, real cohort from the healthcare system with data collected during daily clinical practice, so the selection bias was minimal. Also, the same data collection system was used in all of the primary healthcare centres and PPI use was captured as directly observed therapy. Analytical were performed at the same laboratory. Participants were between 15 and 100 years old with a similar percentage of men and women and we did not exclude participants either because of their age or for any other reasons. There was 8 years of follow-up and we defined incident CKD as an eGFR< 60 ml/ min/1.73 m^2^ and/or UACR ≥ 30 mg/g, in > 2 determinations in a period > 3 months according to KDOQI definition. Moreover, we use statistical methods that allow us to control the methodological problems not contemplated in other studies, such as the existence of non-proportional risks, individual heterogeneity, both constant and time-varying, and the presence of unobservable confounders. Furthermore, we use statistical methods that allow us to control the methodological problems not contemplated in other studies, such as the existence of non-proportional risks, individual heterogeneity, both constant and time-varying, and the presence of unobservable confounders.

This study shows significant association between the use of PPIs and increased risks of CKD. Although no causal relationship has been proven, health providers should consider whether PPI therapy is indicated for their patients and chronic use of PPIs should be avoided and withdrawn in the absence of indications. Given the association with kidney disease, serum creatinine levels should probably be monitored in patients using PPIs, especially those using high doses.

## Conclusions

In this study, we found that use of Proton Pump Inhibitors is associated with an increased risk in the development of CKD, especially after a total exposure time of more than three months and if high doses are used.

The results of our study help us to understand the association between PPI use and the renal side effects observed. They suggest a need for the judicious, reasoned prescription of this drug at minimal doses, limited to the necessary exposure time during which renal function must be monitored.

Although cause and effect cannot be determined with an observational study, providers should consider whether PPI therapy is indicated for the individuals. Careful monitoring of kidney function while on PPI use and cessation of PPIs when there is no clear indication for use might reduce the population burden of CKD.

## Abbreviations

95% CI: 95% Credibility interval
ACEI: Angiotensin-converting- enzyme inhibitor
AG: Andersen-Gill
AIN: Acute interstitial nephritis
ARB: Angiotensin receptor blocker
ARF: Chronic renal failure
BMI: Body mass index
CKD: Chronic kidney disease
DM2: Type 2 diabetes
eGFR: Estimated glomerular filtration rate
g: gram
HDL: High density lipoproteins cholesterol
HR: Hazard Ratio
IAS: Institute of Health Care (IAS)
INLA: Integrated Nested Laplace Approximation
m: metre
mg: milligram
min: minute
ml: millilitres
MS: Metabolic syndrome
NSAID: Nonsteroidal anti-inflammatory drug
PC: Penalising complexity priors
PPI: Proton Pump Inhibitor
UACR: Urinary albumin level to creatinine level
